# Successful Management of a Neglected Talonavicular Joint Dislocation Using Corrective Osteotomy and Subtalar Fixation: A Case Report

**DOI:** 10.7759/cureus.68470

**Published:** 2024-09-02

**Authors:** Gursimran Singh, Nareshkumar Dhaniwala, Kiran M Saoji, Anmol Suneja, Ashutosh Lohiya

**Affiliations:** 1 Department of Orthopaedics, Jawaharlal Nehru Medical College, Datta Meghe Institute of Higher Education and Research, Wardha, IND

**Keywords:** post-traumatic deformity, joint realignment, orthopedic case report, subtalar fixation foot surgery, corrective osteotomy, neglected dislocation, talonavicular joint dislocation

## Abstract

The complete loss of articular relationships between the talus and navicular bone is known as talonavicular joint (TNJ) dislocation. Medial dislocation of the TNJ is more common than lateral dislocation. Lateral dislocation is usually associated with a fracture of the calcaneocuboid joint. Surgeons encounter difficulties when treating these dislocations. It occurs following high-energy trauma and is managed with immediate closed reduction of the joint and immobilization, but some complicated cases require open reduction and fixation to achieve alignment, strength, and function. This case report describes a six-month-old neglected case of TNJ dislocation managed with corrective osteotomy and subtalar fixation. Fusion of the talonavicular and talocalcaneal joints was performed. This procedure had a satisfactory outcome, as the patient was completely relieved of pain.

## Introduction

Subtalar dislocations account for 1% of all dislocations and 15% of all peri-talar injuries. It is more common in men than women. Neglected talonavicular joint (TNJ) dislocations represent one of the more rare and challenging problems in orthopedics because of the complex anatomy of the midfoot, and the long-term potential for major deformity and dysfunction if left untreated [1]. They are usually high-energy injuries - for example, the sequelae of a motor vehicle accident or a fall. If the diagnosis of the dislocation is missed, it manifests as chronic pain, instability, and major functional impairment. Traditional strategies for the management of acute TNJ dislocations have focused on early reduction and immobilization, but the approach to chronic cases that have been neglected is much less well-defined [2].

The neglected TNJ dislocations are rather challenging to handle because, with time, there are adaptive alterations in surrounding bony and soft tissue structures due to the neglected dislocation [2,3]. These changes make reduction and joint realignment more complicated. Corrective osteotomy (medial and lateral swivel osteotomies) and subtalar fixation are new frontiers in surgical management for dealing with these challenges [4]. Corrective osteotomy is only done if a trial of open reduction of the joint fails. Corrective osteotomy realigns the bones back to proper anatomy, and subtalar fixation helps to lock this in place while healing takes place [3].

The duration from injury to surgery greatly affects the prognosis. Better recovery is seen in cases managed immediately after injury, whereas in chronic cases, joint arthritis develops and needs more surgical exploration, which delays recovery. This case report presents the successful management of a six-month-old neglected post-traumatic TNJ dislocation using a combination of corrective osteotomy and subtalar fixation. 

There are different treatment modalities available, like closed reduction and cast application or Kirschner wire fixation, open reduction and internal fixation with screws or plates, and open reduction and external fixation. In our case, open reduction of the talus head was attempted, but it could not be achieved, so osteotomy of the talus head was done, and reduction of the navicular was done, followed by internal fixation. This case report underlines, to bring into the limelight, a surgical technique, its postoperative outcomes, and the possibility of it being a workable treatment option for similar cases soon. 

## Case presentation

A 20-year-old male presented to our hospital with complaints of persistent pain and a hard lump on the medial aspect of his right ankle for six months. The patient was alright six months ago when he had a road traffic accident, after which he started experiencing severe pain in his right ankle and was taken to a local hospital, where an X-ray was done, and he was treated with a below-knee slab for one week and later a cast for one month. On follow-up, the patient was counseled that the pain in his foot would subside as there was no fracture seen in the radiograph done at that time. Despite these treatments, the patient did not experience pain relief but continued walking with a limp. He sought further treatment at our hospital after several months.

**Figure 1:**
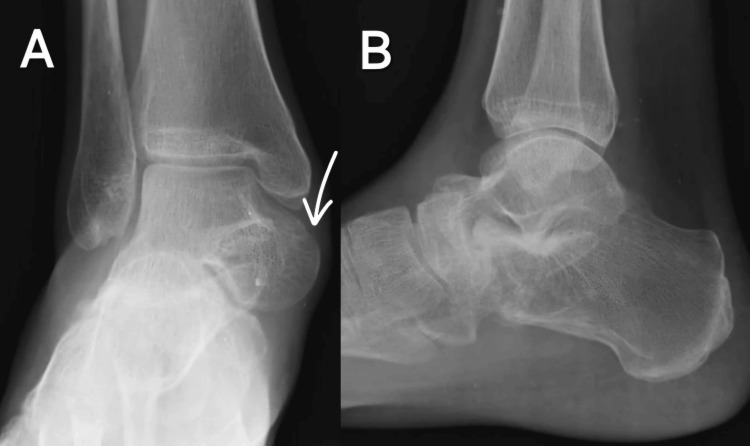
Pre-operative radiograph of the right ankle in (A) anteroposterior and (B) lateral views The white arrow shows subluxation of the talus from the ankle joint, with the navicular completely dislocated laterally, exposing the head of the talus.

Upon admission, a thorough clinical examination was conducted. Radiographic evaluation, including anteroposterior and lateral views of the right ankle, revealed subluxation of the talus from the ankle joint, with the navicular completely dislocated laterally, exposing the head of the talus (Figure 1).

**Figure 2:**
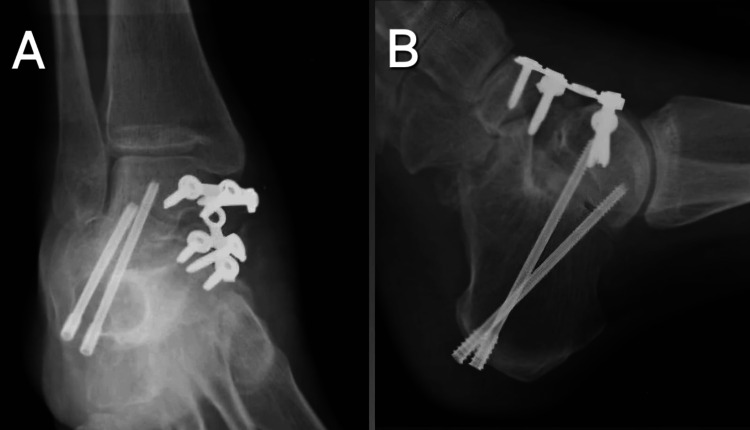
Post-operative radiograph of the right ankle in (A) anteroposterior and (B) lateral views

Given the severity of the condition, surgical management was planned. The patient was taken for the procedure, and through an anteromedial approach, the head and neck of the talus were excised. The articular surface of the talus was found to be intact. The navicular was reduced and aligned with the remaining talus. Fusion was done at the TNJ and stabilized using a four-hole plate fixed with three screws in the nonarticular part of the talus and three screws in the navicular. Additionally, via a sinus tarsi approach, the subtalar joint articular cartilage was excised, and the talocalcaneal joint was fused. The talocalcaneal joint was stabilized with two Herbert screws, securing the calcaneum to the talus (Figure 2).

Postoperatively, the patient had his sutures removed after 12 days. At the three-month follow-up, he demonstrated a good range of painless ankle movements, indicating a successful outcome of the surgical intervention.

## Discussion

The midtarsal joints include the talonavicular and calcaneocuboid joints. Talonavicular dislocations are very rare. Diagnosis is made with plain radiographs and computed tomography scans if required [4]. Hindfoot valgus and forefoot adduction are common deformities that occur when such cases are left untreated. These deformities result in instability, altered biomechanics, and chronic pain over the affected bone and joint. Late complications include avascular necrosis of the talus, osteoporosis, and post-traumatic arthritis [5,6].

Management of TNJ dislocation is challenging; deciding factors include the time from the injury and any associated fractures. Acute injuries are treated with closed reduction and cast application, whereas old cases or those where closed reduction has failed are treated with open reduction [6]. Further subtalar fixation may be required if the hindfoot is unstable. This not only prevents the dislocation from recurring but also provides additional stability to the subtalar joint [3].

Neglected TNJ dislocation requires a versatile surgical approach to address both complex deformities and functional impairments that arise over time. Corrective osteotomy plays a very important role in aligning the bones of the foot to their original position and restoring proper foot mechanics. There have been studies that showed that isolated TNJ dislocations can be managed with subtalar arthrodesis, and the patient was completely pain-free with good weight-bearing postoperatively [7]. Some studies also showed that six-week-old cases of such dislocations, managed with open reduction and k-wire fixation followed by cast application, had good clinical outcomes [8]. Previous studies have proved that closed reduction under general anesthesia, followed by slab application, is the treatment of choice for patients presenting early after injury. Cast application and compression bandages should be avoided, as this may lead to compartment syndrome [9]. Comparing the previous studies to our case, it is found that lateral dislocation of the TNJ mostly needs operative management, as closed reduction can be unsuccessful [10]. In this case study, osteotomy of the dislocated talus bone was done, followed by subtalar fixation, which resulted in pain-free movements and weight-bearing.

## Conclusions

TNJ dislocations are extremely rare, and very little literature is available on management options. The primary goals in treating these patients include pain relief, deformity correction, and restoration of joint function. Old, neglected cases pose a challenge for management. In this case, a trial of corrective osteotomy and subtalar fixation was performed. Subtalar fixation was achieved with plating, and successful results were obtained. The patient was completely relieved of pain and had no difficulty in weight-bearing. As this technique provides stable joint function and pain-free ankle movements, it should be considered in challenging cases of TNJ dislocation where complete reduction of the joint cannot be achieved.
